# Rare Case of Concomitant Pulmonary Tuberculosis and Cytomegalovirus Infection in an Immunocompetent Patient

**DOI:** 10.1155/crdi/5580312

**Published:** 2025-10-30

**Authors:** Theeb Osama Sulaiman, Jamal Sajid, Issam A. Al-bozom, Abdullah Arshad, Sara Khaled Sayed, Irfan Ul-haq

**Affiliations:** ^1^Pulmonology Department, Hamad General Hospital, Hamad Medical Corporation, Doha, Qatar; ^2^Medicine Department, Hamad General Hospital, Hamad Medical Corporation, Doha, Qatar; ^3^Laboratory Medicine and Pathology Department, Hamad General Hospital, Hamad Medical Corporation, Doha, Qatar; ^4^Qatar University, Doha, Qatar

**Keywords:** cytomegalovirus, immunocompetent, lung mass, tuberculosis

## Abstract

Cytomegalovirus (CMV) and tuberculosis (TB) are two distinct infectious diseases that can cause significant health complications and may share clinical manifestations, complicating diagnosis and management. Herein, we present a case report of an immunocompetent patient with concurrent pulmonary CMV and TB infections, highlighting the rare occurrence of dual infection with TB and CMV in an otherwise healthy individual. An enhanced awareness among healthcare professionals about the potential for concurrent infections can improve diagnostic accuracy and patient care in similarly challenging cases.

## 1. Introduction

Tuberculosis (TB) is a notifiable disease in nearly all countries worldwide and remains a significant global health concern, especially in developing regions [1]. Although TB can affect any system, the lungs are the primary site of infection. Pulmonary TB presents with a wide range of clinical symptoms and diverse radiological findings on chest computed tomography (CT) scans [2]. Severe cases are more frequently observed in immunocompromised individuals, although immunocompetent individuals are not entirely spared. Cytomegalovirus (CMV), known as human herpesvirus 5, is a double-stranded DNA virus that establishes latency within myeloid lineage cells. Reactivation of CMV typically occurs in immunosuppressed individuals [3]. While primary CMV infection is often asymptomatic, it can present with a mononucleosis-like syndrome, characterized by fever, pharyngotonsillitis, and lymphadenopathy [4]. CMV reactivation can involve multiple organ systems, including the lungs, and tends to manifest more severely in immunocompromised patients, whereas it is generally milder in immunocompetent hosts. Both TB and CMV are significant pathogens capable of causing substantial morbidity and mortality, particularly in immunocompromised individuals. The concurrent occurrence of these infections in an immunocompetent patient is exceedingly rare, making it a diagnostic challenge. However, this case highlights the importance of considering the possibility of simultaneous TB and CMV infection, even in individuals with no apparent immunosuppression. Recognizing this rare but potential coinfection is crucial for timely diagnosis and appropriate management. This report presents the first known case of concurrent TB and CMV infection in a previously healthy young male.

## 2. Case Presentation

A 45-year-old Asian man, with no notable medical history, presented with a 3-week history of dyspnea and cough. Initially dry, the cough became productive with yellowish sputum. He also experienced an unintentional weight loss of 7 kg over the last 3 months and reported a decreased appetite. However, he denied symptoms such as night sweats, chest pain, palpitations, fever, chills, or recent exposure to illness. Despite completing a course of antibiotics, his symptoms persisted. The patient is a nonsmoker, works as a mechanic, and has no history of recent travel or occupational hazardous exposures. He also denies alcohol or illicit drug use and reports no high-risk sexual behaviors.

On presentation to the Emergency Department (ED), his vital signs were as follows: blood pressure of 119/89 mmHg, heart rate of 99 beats per minute, respiratory rate of 22 breaths per minute, temperature of 37.2°C, and oxygen saturation of 95% while breathing through a nasal cannula at 2 L per minute. Physical examination revealed decreased air entry in the right lung, accompanied by coarse crackles. The remainder of the physical examination was unremarkable.

Laboratory investigations yielded the following results: white blood cell count of 24.8 × 10^9^/L (normal: 4.0–11.0), hemoglobin of 11.3 g/dL (normal: 13.5–17.5 for males; 12.0–15.5 for females), mean corpuscular volume (MCV) of 83 fL (normal: 80–100), and platelets at 399 × 10^9^/L (normal: 150–400). Eosinophils were 0.1 × 10^9^/L (normal: 0.0–0.5), D-dimer was markedly elevated at 3.91 mg/L (normal: < 0.5), creatinine was 239 μmol/L (normal: 60–110), and urea was 11.5 mmol/L (normal: 2.5–7.8). Sodium was 134 mmol/L (normal: 135–145), and potassium was 3.9 mmol/L (normal: 3.5–5.0). Liver enzymes were within normal limits with ALT at 23 IU/L and AST at 17 IU/L (normal: < 40 for both), and bilirubin was 0.4 mg/dL (normal: 0.1–1.2). Random blood glucose was 92 mg/dL (normal: 70–140), and HbA1c was 5.5% (normal: < 5.7%). Inflammatory markers were significantly elevated, with CRP at 305 mg/L (normal: < 5) and procalcitonin at 31.7 ng/mL (normal: < 0.05).

**Figure 1:**
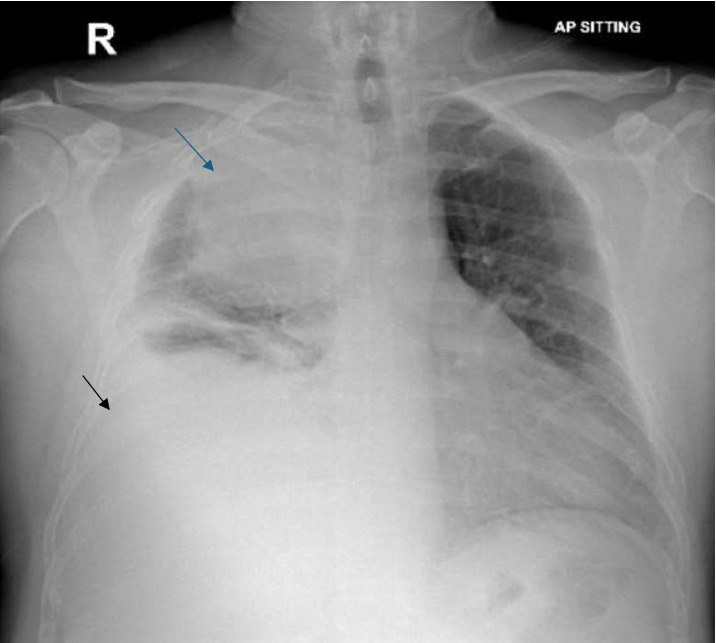
Chest X-ray reveals a homogeneous opacity in the right upper mid-zone, associated with silhouetting of the right heart border (blue arrow), accompanied by moderate right-sided pleural effusion (black arrow).

**Figure 2:**
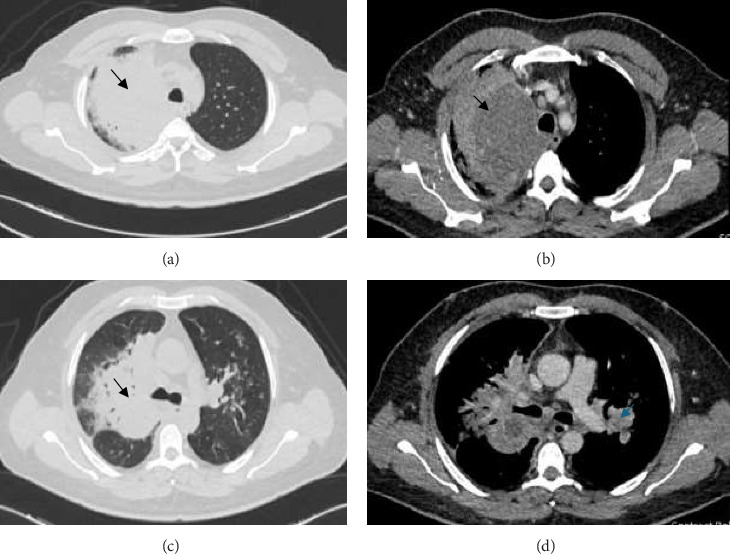
(a–c) Imaging reveals a large, ill-defined, heterogeneously enhancing mass in the upper lobe, peribronchial region, measuring 10.9 × 8.6 × 9 cm, with central hypodensities indicative of necrotic changes. The mass is causing near-complete occlusion of the superior segmental bronchus (black arrows). (d) Multiple enlarged mediastinal lymph nodes; the largest node is in the left hilar region, measuring 2.8 cm in short axis, appearing necrotic with no definitive calcifications (blue arrow).

**Figure 3:**
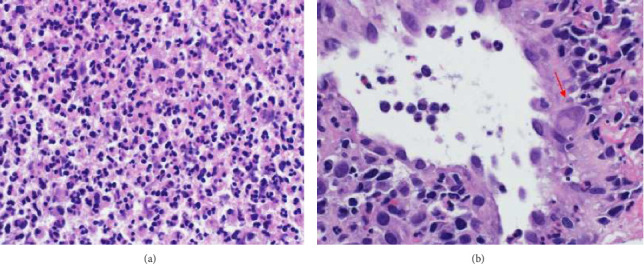
Pathological description: Histopathological examination of the core lung biopsy revealed lung tissue totally replaced by necrotic and inflammatory infiltrate. The inflammatory infiltrate predominantly consists of sheets of neutrophils forming microabscesses (a). Rare intracytoplasmic cytomegalovirus inclusions are seen in the endothelial cells of the blood vessels (b) confirmed by immunohistochemical stains to CMV. Figure legends: (a) Microscopic sections showing microabscess formation replacing lung tissue (H&E ×  400). (b) Light microscopic examination showing CMV inclusion within the endothelial cells (red arrow) (H&E × 400).

**Figure 4:**
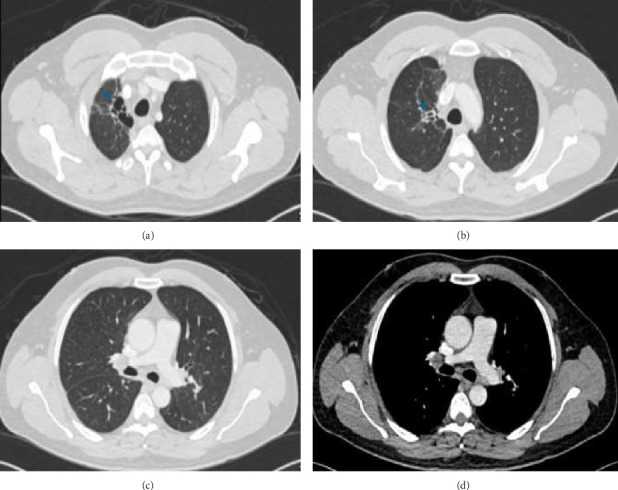
(a–c) Interval resolution of previously seen consolidative changes and mass-like appearance in the right upper lobe and perihilar region, with residual traction bronchiectasis and multiple fibrotic bands in the apical segment (blue arrow). (d) Significant decrease in the size of previously seen multiple enlarged mediastinal lymph nodes.

A chest X-ray revealed a large homogeneous opacity in the right upper mid-zone (Figure 1). Given the elevated creatinine and D-dimer levels, a ventilation-perfusion (V/Q) scan was performed, which ruled out pulmonary embolism. The patient was admitted for intravenous fluid administration and initiated on ceftriaxone and azithromycin. Microbiological evaluation revealed pulmonary TB with a positive sputum TB PCR, prompting the initiation of first-line anti-tuberculous therapy, including isoniazid, rifampin, pyrazinamide, and ethambutol, with a planned treatment duration of 6 months. This diagnosis was later confirmed by positive sputum culture for *Mycobacterium tuberculosis*. Drug susceptibility testing (DST) was performed on the culture isolate using the BACTEC MGIT 960 system, and breakpoints were interpreted according to the Clinical and Laboratory Standards Institute (CLSI) guidelines. The isolate was found to be sensitive to all first-line agents, including isoniazid, rifampin, ethambutol, and pyrazinamide. After 5 days, creatinine levels normalized with intravenous fluids, and a chest CT scan was performed to further characterize the right upper lung lesion and confirmed a large right upper lobe mass (Figure 2). Due to the suspicious nature of the mass, a CT-guided lung biopsy was performed to rule out concurrent malignancy. Histopathological examination confirmed CMV infection (Figure 3). CMV PCR from the serum returned positive with a viral load of 6220 IU/mL. The patient was started on intravenous ganciclovir at a dose of 5 mg/kg, with weekly CMV PCR monitoring. After 17 days of intravenous ganciclovir, the patient was transitioned to oral valganciclovir to complete total duration of 3 weeks of antiviral therapy. Given the rare occurrence of concurrent CMV infection and pulmonary TB, additional blood tests were conducted to exclude underlying acquired or congenital immunodeficiency. The results demonstrated a negative HIV test, normal immunoglobulin levels including IgG subclasses, a negative ANA, and normal lymphocyte subsets. The patient was discharged following clinical improvement. A follow-up high-resolution CT scan 2 months later showed resolution of the right upper lobe consolidation and mass-like appearance, with residual traction bronchiectasis and scarring (Figure 4).

## 3. Discussion

Pneumonia is a clinical condition marked by fever, cough, and alveolar infiltration with purulent exudate caused by infection with a microbial pathogen. It may be due to bacterial, viral, or fungal infections, and the responsible organism is identified in fewer than half of the cases [5]. TB and CMV infections are diseases that particularly affect some of the world's poorest populations and are caused by the microbes *Mycobacterium tuberculosis* and CMV, respectively [4]. Multiple risk factors have been identified for both infections. These include human immunodeficiency virus (HIV) infection, the use of immunosuppressive drugs, diabetes mellitus, nutritional deficiencies, post solid organ or bone marrow transplantation, and malnutrition [6, 7]. Despite these associations, the majority of individuals with TB globally are immunocompetent. Additionally, CMV infections have also been documented in immunocompetent patients [6].

Human CMV is a double-stranded DNA virus within the Herpesviridae family, which commonly infects humans. CMV is transmitted via direct contact with infected body fluids or vertically from mother to fetus [8]. CMV infection can involve multiple organ systems. It can affect the gastrointestinal system causing CMV colitis, esophagitis, and gastroenteritis. Ocular involvement with retinitis, hepatic involvement as hepatitis, cardiovascular involvement such as vascular thrombosis or myocarditis and Pulmonary complications include CMV pneumonia and pneumonitis. CMV infections are typically asymptomatic or present with mild symptoms in immunocompetent patient [9]. Both pulmonary TB and CMV pneumonia can present clinically with wide variety of symptoms including cough, dyspnea, hemoptysis, and weight loss. The clinical manifestations are predominantly observed and more severe in immunocompromised individuals, in the radiological part, Common findings in CMV pneumonia are mixed alveolar-interstitial infiltrates such as ground-glass opacity, consolidation, bronchiectasis and interstitial reticulation. [10], mass-like infiltrates [11], or cavitary lung mass [12]. The radiographic findings in our patient were worrisome for lung cancer. For this reason, CT-guided lung biopsy was performed despite diagnosis of pulmonary TB obtained though sputum sample.

There are similarities in the host immunological response to both CMV and *Mycobacterium tuberculosis*, and the epidemiological overlap between the two diseases is generating considerable interest but is not fully understood. Areas of overlap include humans being the primary host, similar risk factors, and both organisms being able to remain latent in host cells for years before reactivation [13]. In low-income and middle-income countries, 85%–95% of people are CMV-seropositive by age 56 years, and most are infected by age 1 year [14]. CMV affects the immune system through CMV-specific memory CD4+ and CD8+ T-cell activation [15]. Primary CMV infection is characterized by profound expansion of antigen-specific CD8+ and CD4+ T cells and expanded NK cells which can display inappropriate homing to tissue infected with other pathogens and lower IFN-γ secretion in response to pathogens. CMV infection also drives the expansion of CD94/NKG2C NK cells, and these cells play an important role in the control of viral replication [16]. A study involving South African infants found that those who developed TB had higher CMV-specific IFN-γ responses compared to those who did not develop TB. Additionally, CMV-positive infants were observed to contract TB earlier than CMV-negative infants [17]. Other research has indicated that individuals with positive CMV-specific IgG antibodies face a higher risk of both active TB [18] and latent TB (odds ratio: 2.94; 95% CI: 1.19–7.28; *p*=0.02) [19]. Furthermore, a study revealed that children with elevated CMV loads are at a significantly increased risk of developing TB disease [15].

Different hypotheses have been proposed to describe how CMV infection increases the risk of TB infection. One study included those African infants with CMV-positive who progressed to TB disease in the South African cohort and found they had lower expression of CD94 and NKG2C (KLRD1 and KLRC3) transcripts and a lower frequency of NK cells [17]. Another hypothesis is based on the synthesis of IFN-α and IFN-β by the host cells after exposure to CMV. Excessive type I IFNs have been linked to TB disease exacerbation via an eicosanoid imbalance, whereby necrotic, as opposed to apoptotic, cell death is induced, resulting in subsequent bacterial escape and further cellular infection [20]. Additionally, elevated levels of tumor necrosis factor-alpha (TNF-α) and interleukin-6 (IL-6) were associated with both latent TB infection and prior CMV exposure, as indicated by high CMV IgG titers. This suggests a potential immunological link between latent TB and CMV infection through shared proinflammatory pathways [19].

Coinfection of pulmonary CMV pneumonia and pulmonary TB has been documented in a few case reports [21, 22], all involving immunocompromised patients. In contrast, our patient was immunocompetent, with no chronic medical conditions, negative HIV tests, normal immunoglobulin levels, and normal lymphocyte subset analysis. This represents the first reported instance of simultaneous CMV pneumonia and pulmonary TB in an immunocompetent individual.

## Data Availability

The datasets used and/or analyzed during the current study are available from the corresponding author upon reasonable request.
